# Active and Passive Immunization Protects against Lethal, Extreme Drug Resistant-*Acinetobacter baumannii* Infection

**DOI:** 10.1371/journal.pone.0029446

**Published:** 2012-01-10

**Authors:** Guanpingshen Luo, Lin Lin, Ashraf S. Ibrahim, Beverlie Baquir, Paul Pantapalangkoor, Robert A. Bonomo, Yohei Doi, Mark D. Adams, Thomas A. Russo, Brad Spellberg

**Affiliations:** 1 Division of Infectious Diseases, Los Angeles Biomedical Research Institute at Harbor–University of California Los Angeles (UCLA) Medical Center, Torrance, California, United States of America; 2 Division of General Internal Medicine, Los Angeles Biomedical Research Institute at Harbor–University of California Los Angeles (UCLA) Medical Center, Torrance, California, United States of America; 3 David Geffen School of Medicine at University of California Los Angeles (UCLA), Los Angeles, California, United States of America; 4 Departments of Medicine, Pharmacology, and Molecular Biology and Microbiology, Louis Stokes Cleveland Department of Veterans Affairs Medical Center, Case Western Reserve University, Cleveland, Ohio, United States of America; 5 Division of Infectious Diseases, University of Pittsburgh Medical Center, Pittsburgh, Pennsylvania, United States of America; 6 Department of Genetics and Center for Proteomics and Bioinformatics, Case Western Reserve University, Cleveland, Ohio, United States of America; 7 Veterans Administration Western New York Healthcare System, Division of Infectious Diseases, State University of New York, Buffalo, New York, United States of America; University of Ottawa, Canada

## Abstract

Extreme-drug-resistant (XDR) *Acinetobacter baumannii* is a rapidly emerging pathogen causing infections with unacceptably high mortality rates due to inadequate available treatment. New methods to prevent and treat such infections are a critical unmet medical need. To conduct a rational vaccine discovery program, OmpA was identified as the primary target of humoral immune response after intravenous infection by *A. baumannii* in mice. OmpA was >99% conserved at the amino acid level across clinical isolates harvested between 1951 and 2009 from cerebrospinal fluid, blood, lung, and wound infections, including carbapenem-resistant isolates, and was ≥89% conserved among other sequenced strains, but had minimal homology to the human proteome. Vaccination of diabetic mice with recombinant OmpA (rOmpA) with aluminum hydroxide adjuvant markedly improved survival and reduced tissue bacterial burden in mice infected intravenously. Vaccination induced high titers of anti-OmpA antibodies, the levels of which correlated with survival in mice. Passive transfer with immune sera recapitulated protection. Immune sera did not enhance complement-mediated killing but did enhance opsonophagocytic killing of *A. baumannii*. These results define active and passive immunization strategies to prevent and treat highly lethal, XDR *A. baumannii* infections.

## Introduction

Antibiotic resistance is recognized as one of the greatest threats to human health on the planet [1], [2], [3], [4], [5]. In the last decade, *Acinetobacter baumannii* has emerged as one of the most common and highly antibiotic-resistant pathogens in the United States (US) and throughout the world [6], [7], [8]. Indeed, 50–70% of *A. baumannii* clinical isolates are now extensively drug resistant (XDR; i.e. resistant to carbapenems and all other antibiotics except colistin or tigecycline), reflecting a >15-fold increase in just the past 10 years [9], [10], [11], [12], [13]. Infections caused by XDR *A. baumannii* are associated with prolonged hospitalization, tremendous health care costs, and high rates of death despite treatment [6], [8], [12], [14], [15], [16], [17]. Even more concerning is the increasing resistance of *A. baumannii* to both colistin and tigecycline [8], [15], [18], [19], [20]. Such pan-drug resistant (PDR) *A. baumannii* infections are resistant to every FDA approved antibiotic, and are hence untreatable.

Since risk factors for *A. baumannii* infections are understood [21], [22], [23], [24], [25], vaccination of acutely at-risk patients is a promising method to prevent such infections, and antibody-based immunotherapy has promise to improve outcomes from infection. To identify a lead antigenic target for active and passive immunization against *A. baumannii*, a rational screening mechanism was used to identify a candidate vaccine. OmpA was found to be a predominant target of humoral immunity during sublethal *A. baumannii* infection in mice. Recombinant OmpA was an effective vaccine immunogen, protecting mice against lethal infection, and also induced protective antibodies when administered as passive immunization against lethal *A. baumannii* infection.

## Results

### Specific anti-*A. baumannii* antibodies are generated during infection in mice

**Figure 1:**
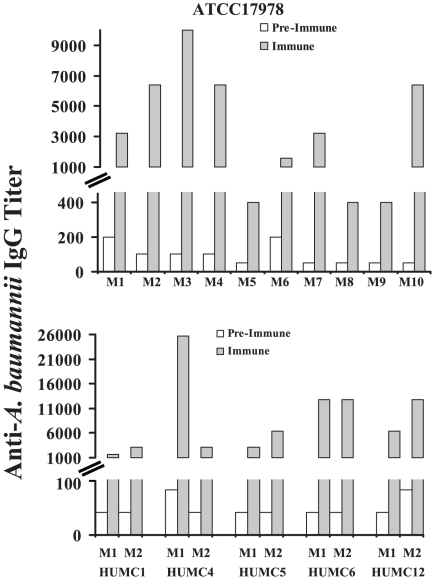
*A. baumannii* infection induces specific humoral immune response. Ten mice were infected with ATCC 17978 (top) and 2 mice each were infected with clinical isolates from Harbor-UCLA Medical Center (HUMC) (bottom). Paired pre-immune & immune serum IgG anti-*A. baumannii* cell membrane protein titers are shown. M1 = mouse 1; M2 = mouse 2.

As a basis for identifying lead antigenic candidates for vaccine development, the humoral immune response to surface proteins from *A. baumannii* was determined after natural infection. Individually marked Balb/c mice were bled via tail-vein nicking to determine baseline, pre-immune anti-*A. baumannii* cell membrane protein antibody titers. Mice were then infected via the tail-vein with survivable inocula (10^6^) of six clinical isolates of *A. baumannii*, five of which were carbapenem resistant (Table 1 and Table S1). Two weeks post-infection, paired immune sera were obtained from the mice. ELISA of paired pre-immune vs. immune sera confirmed that mice infected with all of the strains generated substantial increases (10–100-fold) in anti-*A. baumannii* cell membrane protein IgG-antibody titers by 2 weeks post-infection (Figure 1).

**Table 1:** Bacterial Strains.*

Strain	Strain Type	Source	Carbapenem Resistant?	Comments
ATCC 17978	ST112	ATCC; cerebrospinal fluid isolate	No	Isolated in 1951 from a 4 month old with fatal meningitis [54]
HUMC1	ST206	HUMC, blood and sputum isolate	Yes	Bacteremic VAP
HUMC4	ST208	HUMC, deep endotracheal aspirate	Yes	VAP
HUMC5	ST208	HUMC, bronchoalveolar lavage	Yes	VAP
HUMC6	ST208	HUMC, sputum	Yes	VAP
HUMC12	ST208	HUMC, wound infection	Yes	Infected diabetic stump wound

*HUMC = clinical isolates from in-patients at Harbor-UCLA Medical Center in 2009; VAP = ventilator associated pneumonia. Susceptibility results shown in Table S1.

**Figure 2:**
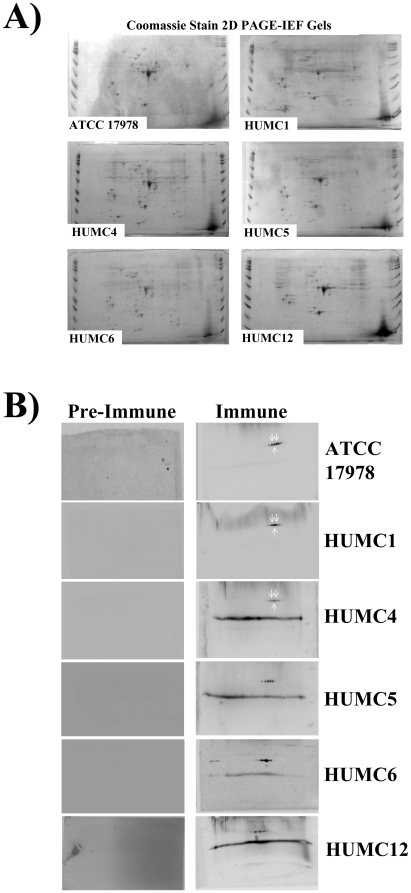
*A. baumannii* infection induces specific anti-rOmpA antibody response. (A) Membrane protein preparations from A. baumanni clinical strains (ATCC 17978 & HUMC1, 4, 5, 6, & 12) were run on 2 D gels stained with Coomassie Blue. (B) Western blots of those 2D gels were stained with paired sera obtained from mice before infection (pre-serum) and after recovery from non-lethal iv infection (post-serum) with *A. baumannii*. 2D gels were run at least twice for all strains, and representative figures are shown. Spots uniquely identified by post-immune serum were seen at conserved locations. Spots selected for protein identification by MALDI-TOF analysis are marked with white arrows—these all contained OmpA.

Having demonstrated a specific humoral immune response to the organism, the immunodominant antigenic target of that response was sought. *A. baumannii* cell membrane protein preparations from all six strains used to infect mice were separated by two dimensional gel electrophoresis and stained by western blot using paired pre-immune and immune sera from the above infected mice. The two dimensional gels demonstrated effective separation by size and isoelectric focusing (IEF) of membrane proteins from all six clinical isolates (Figure 2A). In all cases, post-immune serum identified a limited number of unique spots not recognized by pre-immune serum (Figure 2B).

**Figure 3:**
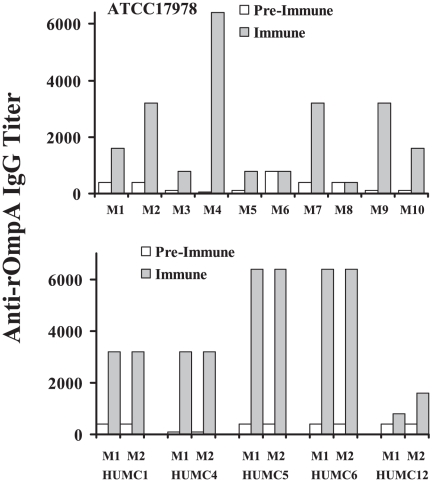
Anti-OmpA IgG antibodies were generated after infection with multiple strains of *A. baumannii*. Ten mice were infected with ATCC 17978 (top) and 2 mice each were infected with HUMC clinical isolates (bottom). Paired pre-immune & immune serum IgG anti-rOmpA cell membrane protein titers are shown.

The same three spots (Figure 2B) were selected for identification by MALDI-TOF analysis across blots from three different *A. baumannii* isolates representing different strain types (Table 1). The protein found in all spots was identified by matrix assisted laser desorption/ionization-time of flight (MALDI-TOF) analysis as OmpA, which is known to be a predominant component of the outer cell membrane of *A. baumannii* [47]. Anti-OmpA antibody titers were determined in paired pre-immune vs. immune sera from mice infected with *A. baumannii*. As for total anti-*A. baumannii* antibodies, anti-rOmpA IgG titers increased in most mice infected with *A. baumanniii* (Figure 3), confirming that OmpA is a target of adaptive humoral immunity post-infection.

### OmpA as a potential vaccine antigen

**Figure 4:**
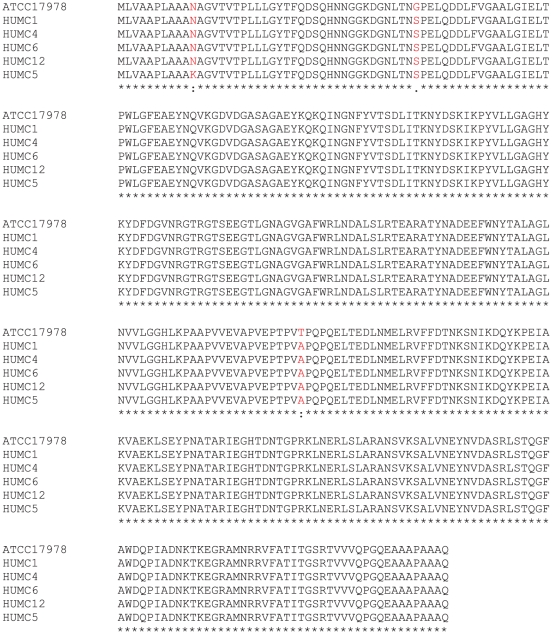
OmpA was highly conserved across clinical isolates of *A. baumannii*. The OmpA gene was sequenced from each strain and the predicted amino acid sequences demonstrated >99% identity.

Ideal antigens for vaccine development should be conserved across clinical isolates and should not be homologous to the human proteome. The *ompA* gene was sequenced in the six clinical isolates used for infection. The predicted protein sequence had 99% identity across all clinical isolates (Figure 4), which were harvested 58 years apart (1951 to 2009) from varied clinical sources (cerebrospinal fluid, lung, blood, wound; Table 1). Alignment against 14 other sequences from *A. baumannii* in PubMed revealed 89% identity across all sequences (Figure S1). In contrast, PubMed BLAST search of the human proteome using the ATCC 17978 OmpA sequence revealed only 7 sequences with minimal homology (E values ranging 0.53 to 6.2). Thus OmpA is conserved across a broad array of clinical isolates of *A. baumannii* but shares minimal homology with human proteins.

To determine *in vivo* efficacy, a lethal infectious model was desired. However, *A. baumannii* bacteremia spontaneously clears in mice unless a host defect is present [39]. Similarly, in our initial pilot experiments, a lethal iv infectious inoculum could not be identified in normal Balb/c mice, unless inocula were so high that they induced overwhelming infection resulting in death within 24 h (e.g., ≥10^9^ bacilli). While neutropenia has been used to make mice susceptible to lethal infection caused by *A. baumannii* [39], [40], [41], neutropenia is a rare clinical risk factor for patients with *A. baumannii* infections [12], [21], [22], [23], [42], [43], [44], [45]. Thus an alternative means to immunocompromise mice was sought. By multivariate analysis, diabetes mellitus has been shown to be a risk factor for acquisition of and worse outcomes from *A. baumannii* infection [23], [24], [46], so a diabetic mouse model of mucormycosis [28] was adapted for *in vivo* study of *A. baumannii* infections. In pilot studies, an inoculum of 2 to 3×10^7^ of strain HUMC1 was found to cause lethal iv infection in diabetic Balb/c mice (data not shown).

**Figure 5:**
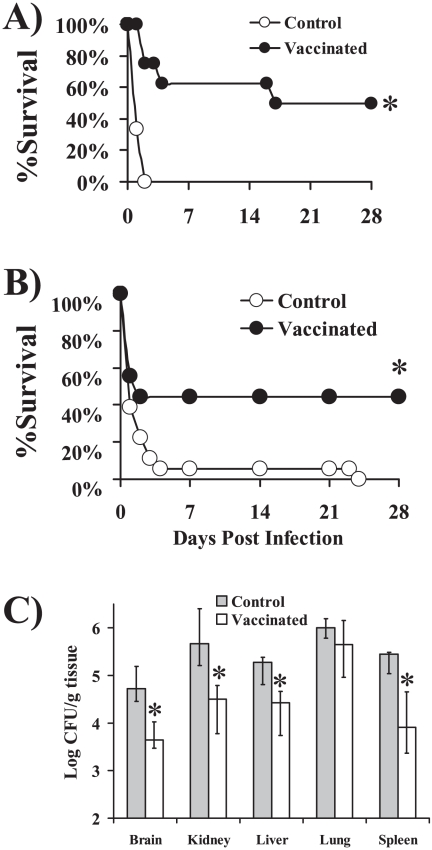
Vaccination with rOmpA protected mice from lethal *A. baumannii* infection in a disseminated sepsis model. A) Survival of retired breeder (>6 mo) diabetic Balb/c mice vaccinated with 3 µg of rOmpA plus aluminum hydroxide (AlOH_3_) adjuvant, or with adjuvant alone (n = 6 adjuvant control and 8 vaccinated) and infected with 2×10^7^ *A. baumannii* HUMC1. B) Survival of juvenile (8–10 weeks, n = 18 mice per group) diabetic Balb/c mice vaccinated with 3 µg of rOmpA plus adjuvant or adjuvant alone and infected with 2×10^7^ *A. baumannii* HUMC1. C) Tissue bacterial burden in vaccinated (3 µg) or control diabetic mice (n = 10 control and 13 vaccinated) infected with 10^7^ *A. baumannii* HUMC1. Median and interquartile ranges are shown. * p<0.05 vs. adjuvant control.

rOmpA was expressed in *E. coli* and purified by nickel-agarose binding to a His tag. In the initial experiment, retired breeder (>6 months old) mice were vaccinated and boosted with rOmpA in 0.1% aluminum hydroxide (Al(OH)_3_). Diabetes was induced after the boost and two weeks later, diabetic mice were infected via the tail-vein with *A. baumannii* HUMC1. Vaccinated mice had significant improvements in survival compared to adjuvant control mice (Figure 5A). The experiment was repeated using juvenile mice and again the vaccine improved survival compared to adjuvant control mice (Figure 5B).

To determine the impact of vaccination on bacterial burden, juvenile mice were vaccinated, made diabetic, and infected as above. On day 2 post-infection (the day the control mice were predicted to die based on the previous experiment), mice were euthanized and organs harvested to determine tissue bacterial burden. Vaccination reduced by approximately 10-fold the tissue bacterial burden in all organs evaluated except for the lungs, which had a non-significant (p = 0.08) 3-fold reduction in bacterial burden (p<0.01 bacterial burden in vaccinated vs. control mice for all other organs) (Figure 5C).

### Antibodies in vaccine-mediated protection

**Figure 6:**
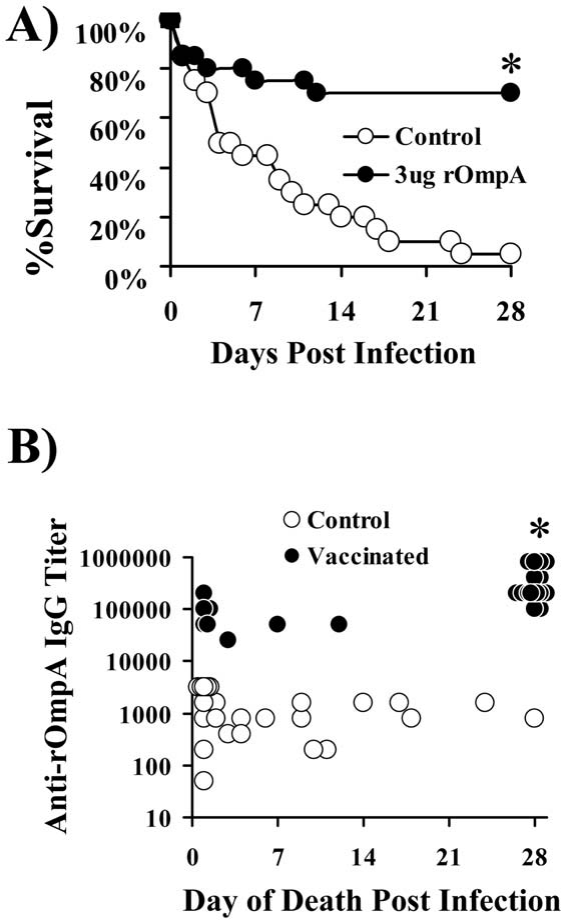
Anti-rOmpA antibody titers correlated with survival in infected mice. A) Survival of juvenile diabetic Balb/c mice vaccinated with 3 µg of rOmpA plus adjuvant or adjuvant alone (n = 20 mice per group from 2 experiments) and infected with 1.4 or 1.6×10^7^ *A. baumannii* HUMC1 in the sequential experiments. The experiments were terminated at 28 days with all remaining mice appearing clinically well. B) Antibody titers of individual vaccinated (n = 26) and control (n = 28) mice vs. day of death.

The relationship between antibody titers and survival in vaccinated mice was evaluated. In two separate experiments, mice were vaccinated with rOmpA plus adjuvant or adjuvant alone, boosted, and antibody titers were determined pre-infection. Vaccination with 3 µg of rOmpA induced marked increases in anti-rOmpA IgG antibody titers compared to control mice (median [range] titers = 204,800 [102,400–409,600] for vaccinated vs. 800 [800–2,000] for adjuvant control mice, p<0.0001). Vaccination again protected mice from lethal infection (note slightly lower inoculum for these experiments, 1.4×10^7^ and 1.6×10^7^ for the repeat experiments, vs. 2×10^7^ and 2.4×10^7^ in the previous survival experiments) (Figure 6A). Antibody titers correlated with survival (Figure 6B) when analyzing both vaccinated and control mice combined (p<0.0001, rho = 0.5) or just analyzing vaccinated mice without control mice (p = 0.001, rho = 0.6 by Spearman Rank test). An IgG titer threshold of ≥204,800 was maximally accurate at distinguishing survivors from non-survivors when analyzing both vaccinated and control mice (96%) or when analyzing just vaccinated mice (85%).

**Figure 7:**
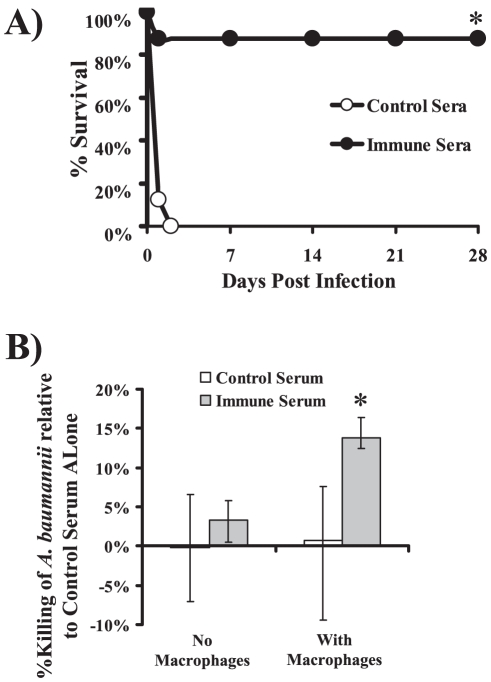
Passive immunization with immune serum from rOmpA-vaccinated mice protected recipient mice from lethal infection. A) Survival of juvenile diabetic Balb/c mice (n = 10 per group) treated ip with immune (from OmpA vaccinated donor mice) or non-immune (from adjuvant treated donor mice) serum 2 hours before tail-vein infection with 2×10^7^ *A. baumannii* HUMC1. The experiments were terminated at 28 days with all remaining mice appearing clinically well. *p = <0.0001 vs. non-immune serum. B) Opsonophagocytic killing of *A. baumannii* HUMC1 by immune (from OmpA vaccinated mice) or control (from adjuvant treated mice) serum incubated without or with RAW 247.6 macrophages. Median and interquartile killing is shown, normalized to the control serum. Results are from 8 to 12 samples per group, from 3 separate experiments. *p<0.05 vs. all other groups.

To confirm the activity of immune antibodies, serum was harvested from donor vaccinated or control mice (rOmpA titers = 1∶409,600 from vaccinated vs. 1∶3,200 from control sera). Diabetic mice were treated ip with 0.5 ml of immune or control serum and infected 2 hours later with *A. baumannii* HUMC1. Mice treated with immune serum had markedly enhanced survival vs. mice treated with control serum (Fig. 7A). To define the mechanism of antibody-induced protection, *A. baumannii* was cultured in the presence of immune vs. non-immune serum. *A. baumannii* numbers doubled or tripled relative to growth controls (absent serum) after 1 hour culture in both immune and non-immune sera at both 10% and 40% (data not shown), excluding complement-mediated killing as a mechanism of protection. Immune serum also did not reduce CFUs relative to control serum (Fig. 7B). However, immune serum did enhance opsonophagocytic killing of *A. baumannii* (Fig. 7B).

## Discussion

Over the past decade *A. baumannii* has emerged to become one of the most antibiotic-resistant causes of infections all over the world. It is critical that new strategies are developed to prevent and treat such infections. Therefore, a rational discovery program was undertaken to identify a candidate antigen for an *A. baumannii*-targeted vaccine. Antigen discovery was based on identification of the immunodominant targets from *A. baumannii* membrane protein preparations following systemic infection. rOmpA was identified as a promising candidate for active and passive immunization based on humoral immunodominance during infection in mice. OmpA was highly conserved across multiple clinical isolates, and shared minimal homology with the human proteome. Substantial efficacy was seen in lethal murine models in immunocompromised, diabetic mice when administered with Al(OH)_3_ adjuvant.

Individual mouse antibody titers correlated with survival and immune serum was effective during passive immunization. It has been previously reported that *A. baumannii* can be resistant to complement-mediated killing [48], [49], however the complement resistance in *A. baumannii* appears to be strain dependent [50]. In a previous study, complement susceptible strains were reported to decrease in quantity by 5 to 10-fold after 1 hour of incubation in serum, whereas resistant strains increased during that hour by a similar amount [50]. In the current study, the *A. baumannii* strains tested doubled or tripled after 1 hour of culture in the presence of serum (immune and non-immune), ruling out a direct complement-mediated effect. Hence, antibodies to OmpA did not overcome the innate resistance of the organism to complement-mediated killing. However, immune serum from vaccinated mice did enhance opsonophagocytic killing of the organism. Collectively, these results confirm that enhanced uptake and killing of *A. baumannii* by antibody-based opsonophagocytosis lead to more effective clearance of *A. baumannii* from tissue. Thus, phagocytic killing of *A. baumannii* can be enhanced by antibodies targeting OmpA.

*A. baumannii* OmpA has been found to have a variety of interesting biological properties in *in vitro* model systems. For example, OmpA has been shown to bind to eukaryotic cells, translocate to the nucleus, and induce cell death [47], [51]. Furthermore, OmpA binding to Factor H may be responsible for the resistance of *A. baumannii* to complement-mediated killing [48], [49]. However, as mentioned, in the current study antibodies targeting OmpA did not overcome serum resistance of the organism. Rather, anti-OmpA antibodies enhanced opsonophagocytic killing of the organism.

Recently, a whole cell, killed *A. baumannii* vaccine was described which protected mice from infection [52]. The investigators prepared crude cell membrane protein preparations and found that the immunologically active components of the whole cell vaccine were found in the cell membrane [53]. The crude membrane preparation contained at least 61 separate proteins, and the resulting mixture protected mice from lethal *A. baumannii* infection. These results underscore the potential for *A. baumannii* vaccines to be effective, and are complementary to the current study, which defines one antigen as a promising lead candidate to develop a recombinant protein based vaccine, as opposed to a crude cell membrane extract. In contrast to the previous study, which found that antibodies were raised against numerous antigens when a crude membrane preparation was used to immunize mice [53], the current study defined humoral immune response after iv infection with viable, pathogenic organisms, rather than immunization with membrane protein preparations. While OmpA was identified as a predominant protein target of humoral immunity after iv infection, the current results cannot exclude a broader immune response to other proteins as well.

In summary, rOmpA is a promising candidate for active and passive immunization to prevent XDR/PDR *A. baumannii* infections. Efficacy has been established at feasible doses with a translatable adjuvant. Use of the vaccine elucidated opsonophagocytic antibodies as the mechanism of adaptive host defense that protected against *A. baumannii* infection. Anti-OmpA antibody titer was identified as a surrogate marker of protection. These results underscore the translational potential of rOmpA as a target for active and passive immunization against this highly antibiotic-resistant, rapidly emerging pathogen.

## Materials and Methods

### Organism and mouse strains

Six clinical isolates of *A. baumannii* were used (Table 1 and Table S1). Five of the strains were resistant to all antibiotics except for colistin. Strain typing was performed by multi-locus sequence typing as previously described [26], [27]. Balb/c mice were used for all experiments. For some experiments, retired breeder mice (>6 mo old) were used, whereas for other experiments juvenile (6–10 weeks old) Balb/c mice were used. Diabetes was induced by intraperitoneal injection of 200 mg/kg streptozotocin in 0.2 ml citrate buffer 10 days prior to infection. Glycosuria and ketonuria were confirmed in all mice 7 days after streptozotocin treatment, as previously described [28].

### Cell Membrane Preparations, Western Blots, 2 Dimensional Gel Imaging, and Protein Identification

*A. baumannii* cell membrane preparations were produced by a modification of a standard, published method [29], [30]. In brief, *A. baumannii* strains were grown overnight at 37°C with shaking in tryptic soy broth (TSB). The bacteria were passaged to mid-log-growth at 37°C with shaking, washed, and the resultant pellet was resuspended in disintegration buffer (7.8 g/L NaH_2_PO_4_, 7.1 g/L Na_2_HPO_4_, 0.247 g/L MgSO4 7.H_2_O+protease inhibitor mix (GE Healthcare, USA)+nuclease mix (GE Healthcare, USA)) and sonicated on ice for 3 periods of 5 min. The unbroken cells were separated by centrifugation at 1,500 *g*. The supernatant was centrifuged for 30 min at 4°C at 4,500 rpm and was passed through a 0.45 µM filter (Milipore, USA) to remove cell debris. An equal volume of ice-cold 0.1 M sodium carbonate (pH 11) was added to the resulting supernatant and the mixture was stirred slowly overnight, on ice. The carbonate treated membrane proteins were collected by ultracentrifugation at 100,000 *g* for 45 min at 4°C, and the membranes were re-suspended in 500 µl H_2_O. Finally, the protein extract was processed with a 2-DE Cleanup Kit (Bio-Rad, USA).

Two dimensional SDS/10%-PAGE gels of *A. baumannii* cell membrane preparations were used to separate proteins by size and isoelectric focusing (IEF), as described by Pitarch et al [31], [32]. For isoelectric focusing (IEF), the Bio-Rad-PROTEIN IEF system was used (Bio-Rad, USA) with 4–7 pH gradient strips (ReadyStrip IPG strips, Bio-Rad, USA). Proteins were solubilized in 8 M urea, 2% (w/v) CHAPS, 40 mM DTT and 0.5% (v/v) corresponding rehydrated buffer (Bio-Rad, USA). The strips were rehydrated overnight and underwent electrophoresis at 250 V for 20 min, 4000 V for 2 h, and 4,000 V for 10,000 V-h, all at room temperature. Prior to the second dimension (SDS-PAGE), the focused IPG strips were equilibrated with buffer I and II for 10 min (ReadyPrep 2-D Starter Kit, Bio-Rad, USA). The proteins were separated on 8–16% Criterion Pre-cast Gel (Bio-Rad, USA) and transferred to immune-Blot PVDF membranes (Bio-Rad, USA). Membranes were treated with Western Blocking Reagent (Roche) overnight and probed with pre-immune or immune *A. baumannii* infected-mice serum. Membranes were washed and incubated with secondary, HRP-conjugated goat anti-mouse IgG (Santa Cruz Biotech, USA). After incubation with SuperSignal West Dura Extended Duration Substrate (Pierce, USA), signals were detected using a CCD camera.

Protein spots of interest were excised and sent to the UCLA W. M. Keck Proteomic Center for identification on a Thermo LTQ-Orbitrap XL mass spectrometer (San Jose, CA) equipped with an Eksigent (Dublin, CA) NanoLiquid chromatography-1D plus system and an Eksigent autosampler. Proteins within the spots were in-gel tryptic digested as described by Shevchenko *et al.* [33], [34]. The eluted peptides were loaded onto a CVC Microtech (Fontana, CA ) 35 mm length, 100 µm ID C18 pre-Trap column and washed for 10 min with 100% Buffer A (2% acetonitrile containing 0.1% formic acid) at a flow rate of 5 µl/min. The peptides were separated on a 15 cm New Objective ProteoPep IntegraFrit column (Woburn, MA) using a flow rate of 300 nl/min. The following elution gradient was used: 0–15 min 0–30% Buffer B (98% acetonitrile containing 0.1% formic acid), 15–20 min 30–80% Buffer B and 20–22 min 80% Buffer B. The column was then re-equilibrated for 13 min with Buffer A. The eluting analytes were sprayed in positive mode into the LTQ-Orbitrap MS using electrospray ionization voltage of 2300 V, capillary voltage of 45 V, tube lens of 130 V, and capillary temperature of 200°C. Information dependent acquisition was performed where the 6 most intense ions were selected in the *m/z* range of 300–1600 using a 60 K resolution FTMS scan and subjecting them to MS-MS using broadband collision induced disassociation of normalized collision energy of 35 and LTQ detection. Peaks were excluded from further MS-MS for a period of 60 sec.

The resulting MS/MS spectra was searched against the *Acinetobacter baumannii* strain ATCC 17978 database (http://gib.genes.nig.ac.jp/single/blast2/main.php?spid=Abau_ATCC17978) using the Matrix Science MASCOT Daemon search engine (Boston, MA). The following search parameters were used: peptide tolerance: ±10 ppm, MS/MS tolerance ±0.3 Da, maximum missed cleavages: 2, fixed modifications: carboxymethyl (C) and variable modifications: deamidization (ND) and oxidation (M). Proteins identified within a particular included those with a minimum of two unique peptides that are ranked as number 1 and with an ion scores with a p<0.05.

### rOmpA Production and Immunization

His-tagged rOmpA (amino acids 2 to 347) was produced in an *Escherichia coli* pQE-32 expression system (Qiagen) as previous described [35], [36]. Briefly, *ompA* was amplified from *A. baumannii* 17978 genomic DNA with primers OmpA-F *CATCACCATGGGATCCTTGTTGCTGCTCCATTAGCT* and OmpA-R *CTAATTAAGCTTGGCTGCAGTTATTGAGCTGCTGCAGGA* and cloned into QE-32 by using In-Fusion 2.0 Dry-Down PCR Cloning Kit, per the manufacturer's instructions (Clontech Laboratories). The 6X-His tagged protein was purified over a Ni-agarose affinity column according to the manufacturer instructions (Qiagen). Endotoxin was removed from rOmpA by using Detoxin Gel Endotoxin Removing Columns (Norgen Biotek, Canada), and the endotoxin level was determined with Limulus Amebocyte Lysate endochrome (Charles River) per manufacturer's instruction. Using this procedure, endotoxin was reduced to 1 to 4 EU per 3 µg dose used for vaccination. Mice were immunized by subcutaneous injection of 3 µg of rOmpA in 0.1% Al(OH)_3_ (Alhydrogel, Brenntag Biosector, Frederikssund, Denmark) in phosphate buffered saline (PBS). Control mice received adjuvant alone on the same schedule. Mice were immunized 5 weeks prior to infection and again 2 weeks prior to infection. Four days after the boost (10 days prior to infection), mice were rendered diabetic as described above.

### Mouse model of infection

*A. baumannii* strains were grown overnight at 37°C with shaking in TSB broth. The bacteria were passaged to mid-log-growth at 37°C with shaking. Cells were washed twice with PBS and resuspended at the appropriate concentration for infection. The final concentration was confirmed by quantitative culturing of the inocula. Mice were infected iv via the tail-vein with sublethal (10^6^) or lethal (targeted 2×10^7^) inocula in PBS. All animal work was conducted after approval by the Institutional Animal Use and Care Committee at the Los Angeles Biomedical Research Institute (project 012447), in compliance with the recommendations in the Guide for the Care and Use of Laboratory Animals of the National Institutes of Health.

Two days after infection (the day on which control mice were anticipated to begin dying), organs were harvested and homogenized in sterile PBS. Homogenized organs from individually marked mice were quantitatively cultured to determine tissue bacterial burden.

### ELISAs

A previously published ELISA assay [37], [38] was adapted for detection of antibodies against *A. baumannii* cell membrane preparations and rOmpA. In brief, ELISA plates were coated with 100 µl per well of 5 µg/ml of rOmpA or cell membrane preparation. Coated wells were blocked with bovine serum albumin, incubated with mouse sera, washed, and stained with goat anti-mouse secondary antibody conjugated with horseradish peroxidase. Wells were washed again and incubated with *o*-phenylenediamine substrate with H_2_O_2_. The color was allowed to develop for 20 min after which the reaction was terminated by adding equal volume of 3N HCl and the optical density (OD) was determined at 490 nm in a microtiter plate reader. Negative control wells received an irrelevant isotype control monoclonal antibody rather than mouse serum. The ELISA titer was taken as the reciprocal of the last serum dilution with an OD reading≥(mean OD of negative control samples+(standard deviation * 2)).

### Complement and Opsonophagocysis Assays

*A. baumannii* HUMC1 was cultured overnight in tryptic soy broth (TSB) at 37°C, passaged to mid-log growth, rinsed, and aliquoted into 96 well microtiter plates. For complement studies, 10% or 40% non-immune or immune sera were added to the wells for 1 hour. Well contents were quantitatively cultured at baseline and again at 1 h. The opsonophagocytic kill assay was based on a modification of a previously used method [25]–[26]. Murine RAW 264.7 macrophage cells (American Type Culture Collection, Rockville, MD) were cultured at 37°C in 5% CO_2_ in RPMI 1640 (Irvine Scientific, Santa Ana, CA) with 10% fetal bovine serum (FBS), 1% penicillin, streptomycin, and glutamine (Gemini BioProducts), and 50 µM β-mercaptoethanol (Sigma-Aldrich, St. Louis, MO). RAW 274.7 cells were activated by 3 days of exposure to 100 nM PMA (Sigma-Aldrich). Activated RAW 264.7 macrophages were harvested after scraping with BD Falcon cell scrapers (Fischer Scientific) and added to the microtiter wells at a 20∶1 ratio of macrophages to bacteria. After a 1 hour incubation with gentle shaking, aliquots from the wells were quantitatively plated in tryptic soy agar (TSA). Colony forming units (CFU) of individual tubes were normalized to the average CFUs in tubes with control serum, and percent killing was calculated as 1−(CFUs from the individual tube/average CFU in tubes with control serum).

### Statistics

Survival was compared by the non-parametric Log Rank test. Antibody titers and bacterial burden were compared with the Wilcoxon Rank Sum test for unpaired comparisons or the Wilcoxon Signed Rank test for paired comparisons, as appropriate. Multiple comparisons were corrected by the Tukey non-parametric test. Correlations were determined by the Spearman Rank test. All statistics were run using Kyplot. Differences were considered significant if the p value was <0.05.

## Supporting Information

Figure S1 **Homology of rOmpA to** ***A. baumannii*** **strains.** OmpA is >99% homologous at the amino acid level across the six clinical isolates of *A. baumannii* used in the current study, including carbapenem-susceptible and carbapenem-resistant strains. C) 14 additional with sequences in Pubmed Genbank.
(TIFF)

Table S1 Susceptibility Testing for Strains Studied.
(DOC)
