# Supplementary material for: Active and Passive Immunization Protects against Lethal, Extreme Drug Resistant-Acinetobacter baumannii Infection
Source: PLoS One. 2012 Jan 10;7(1):e29446. doi: 10.1371/journal.pone.0029446 (PMC3254619; doi:10.1371/journal.pone.0029446)
Supplement: Table S1 — Susceptibility Testing for Strains Studied. (DOC) [file pone.0029446.s002.doc]

**Supplemental Table 1. Susceptibility Testing for Strains Studied**

| **Strain** | **Amikacin** | | **Gentamicin** | | **Aztreonam** | | **Ampicillin/ sulbactam** | | **Pipercillin/ tazobactam** | | **Cefepime** | | **Meropenem** | |
| --- | --- | --- | --- | --- | --- | --- | --- | --- | --- | --- | --- | --- | --- | --- |
| ATCC 17978 | 8 | | 8 | | 16 | | 1/0.5 | | 0.06/4 | | 2 | | 0.25 | |
| HUMC1 | >128 | | >128 | | 64 | | 16/8 | | <128/4 | | 16 | | 32 | |
| HUMC4 | >128 | | >128 | | 32 | | 32/16 | | <128/4 | | 16 | | 8 | |
| HUMC5 | >128 | | >128 | | 32 | | 32/16 | | <128/4 | | 16 | | 8 | |
| HUMC6 | >128 | | >128 | | 32 | | 32/16 | | <128/4 | | 16 | | 8 | |
| HUMC12 | >128 | | >128 | | 32 | | 32/16 | | <128/4 | | 16 | | 4 | |
| **Strain** | | **Imipenem** | | **Ertapenem** | | **Doripenem** | | **Ciproflox-acin** | | **Tigecyc-line** | | **Colistin** | |  |
| ATCC 17978 | | 0.25 | | 4 | | 0.5 | | 0.125 | | 0.25 | | 2 | |  |
| HUMC1 | | 16 | | 128 | | 16 | | >128 | | 4 | | 2 | |  |
| HUMC4 | | 4 | | 32 | | 4 | | 64 | | 4 | | 2 | |  |
| HUMC5 | | 4 | | 32 | | 8 | | 64 | | 4 | | 2 | |  |
| HUMC6 | | 4 | | 32 | | 4 | | 64 | | 4 | | 2 | |  |
| HUMC12 | | 2 | | 16 | | 8 | | 64 | | 4 | | 2 | |  |
